# Supplementary material for: Reconciling discrepant minor sulfur isotope records of the Great Oxidation Event
Source: Nat Commun. 2023 Jan 17;14:279. doi: 10.1038/s41467-023-35820-w (PMC9845385; doi:10.1038/s41467-023-35820-w)
Supplement: Supplementary file 1 — Supplementary Information [file 41467_2023_35820_MOESM1_ESM.pdf]

Supplementary Information -

Reconciling discrepant minor sulphur isotope records of the Great Oxidation Event

Benjamin T. Uveges[1#], Gareth Izon[1], Shuhei Ono[1], Nicolas J. Beukes[2], Roger E. Summons[1]

[1]Department of Earth, Atmospheric and Planetary Sciences, Massachusetts Institute of Technology, 77 Massachusetts Avenue, Cambridge, MA 02139, USA.

[2]DSI-NRF Centre of Excellence for Integrated Mineral and Energy Resource Analysis, Department of Geology, University of Johannesburg, P.O. Box 524, Auckland Park 2006, South Africa.

[#]To whom correspondence may be addressed: Benjamin T. Uveges – buveges@mit.edu

1. Uncertainties Surrounding Fluctuations within the S-MIF record:

1.1 Age Constraints on the Turee Creek Group, Australia

The ubiquitous presence of subdued positive $\Delta^{33}S$ values (~0.5–1.95‰) throughout the Turee Creek Group, extending beyond the demise of S-MIF in South Africa, has been taken as an analytical demonstration of the crustal memory effect (CME)[1,2]. This interpretation, however, is reliant on the correlation between the Kaapvaal and Pilbara-housed quadruple sulphur isotope (QSI) records, which is not universally accepted[3]. In detail, Philippot et al. presented a Re–Os isochron age (2,312.7 ± 5.6 Ma) from the Meterorite Bore Member, which they interpreted as a depositional age[1], consistent with a maximum depositional age inferred from detrital zircon analyses (< 2,340 ± 22 Ma[4]) from the same unit (Fig. 1). More recently, however, the significance of these apparently consistent age constraints has been contested[3]: Here, rather than a depositional age[1], Bekker and colleagues ascribe the Meteorite Bore Re–Os age to a regional-scale hydrothermal overprinting, while citing elevated uranium contents[3,5] to dismiss the detrital zircon-derived ages[4]. In the absence of direct constraint, these authors present a series of arguments from which they infer that the entire Turee Creek succession (~3 km worth of sedimentary rock) was deposited, an order of magnitude faster than existing estimates (2,450–2,200 Ma)[3,6], between 2,450 and 2,420 Ma (Supplementary Fig. 1). Though the merits of these contestations are still actively debated[5,7], if the Turee Creek does not correlate with the Duitschland/Rooihoogte–Timeball Hill formations, then the observed S-MIF may capture the earlier operation of oxygen-free atmospheric photochemistry rather than the expression of a CME. For now, however, in the absence of additional direct age constraints, we note the coherence between independent geochronometers and assume the validity of the ages presented by Philippot et al.[1] and Caquineau et al.[4]. Our modelling results for a Pilbara-specific CME should therefore be viewed as a way to reconcile the Turee Creek group data with the South African data, but are only necessary if these ages stand.

1.2 Examining the Evidence for Multiple Oxygenation Episodes

A series of recent papers have discussed the possibility that the Great Oxidation Event was, in fact, not a single unidirectional event but, rather, proceeded via a series of broad-scale episodes where oxygen dynamics were paced by climatic and/or tectonic drivers[3,8,9]. Here, the dynamic operation of the atmosphere, and specifically its oxygen content, is inferred from the apparent loss and re-emergence of S-MIF within the geological record. Consequently, deciphering Earth's oxygenation trajectory is reliant on our ability to successfully correlate between globally distributed sedimentary successions (Supplementary Fig. 1-2)—A task, itself, plagued by incomplete and contested chronostratigraphic frameworks, not to mention outstanding questions surrounding the atmospheric fidelity of the underpinning MSI records. Indeed, depending on one's favoured interpretation(s), the case could be made for a singular loss of S-MIF at ~2.33 Ga, or upwards of three major oxygenation episodes. Here we discuss some pertinent outstanding questions/uncertainties, providing the general arguments that surround each and directions towards their resolution.

1.2.1 The Loss of S-MIF in Fennoscandia

Warke et al. report quadruple sulphur isotope data from the Fennoscandian Seidorechka and Polisarka Sedimentary formations[10]. Here, based on highly uncertain $\Delta^{36}S/\Delta^{33}S$ slopes, these authors argue for a singular disappearance of S-MIF, signalling the rise of atmospheric oxygen above 10^{-5} of present atmospheric levels (PAL) in the prelude to a Snowball Earth glaciation at around 2.45 Ga[10] (Supplementary Fig. 2). This same Fennoscandian loss of S-MIF was later cited by Poulton et al.[9] as evidence for the first of potentially three hypothesised "oxygenation episodes" originally described by Gumsley et al.[8] that preceded their own recently identified Timeball-Hill-housed S-MIF oscillations (Supplementary Fig. 1). Consequently, while the apparent loss of S-MIF from the Fennoscandian QSI record at *c.*2.4 Ga has the potential to represent a crucial juncture in Earth's oxygenation history, there are several potential issues that require exploration before we can accept this interpretation.

The Limestone Member of the Polisarka Formation features positive $\Delta^{33}S$ and $\Delta^{36}S$ values, representing a direct departure from canonical S-MDF predictions and Archean-like S-MIF systematics alike, wherein $\Delta^{33}S$ and $\Delta^{36}S$ values have opposing signs[11]. Although a combination of linear and orthogonal regressions through a select subset of these data were used to demonstrate their compliance to canonical mass laws, and thus the loss of S-MIF, the 1σ uncertainties associated with the resultant slopes were extreme (± 53.54[10]) and, while statistically permissible, remain non-diagnostic. Extracting the QSI data (i.e., those with both $\Delta^{33}S$ and $\Delta^{36}S$ values) from our database (*n* = 4,669) reveals that less than 4% of the available pre-23. Ga QSI data lie within/above the range reported from the Limestone Member (minimum $\Delta^{36}S$ = 0.213 and $\Delta^{33}S$ = 0.03), and, of these, 67% of the measurements were the product of less precise secondary ion approaches (1σ = $\Delta^{36}S_{Mean}$ ± 0.45‰, $\Delta^{36}S_{max}$ ± 1.42‰). Omitting these secondary-ion-derived data, shows that the QSI systematics of the Limestone Member are only comparable to ~1.2% of the remaining database, thereby demonstrating the peculiarity of the Fennoscandian dataset relative to the wider QSI record. Overlying the Seidorechka Volcanic Formation, the Limestone Member of the Polisarka Formation features an extensive volcanic component, with numerous instances of intercalated mafic and ultra-mafic volcanic rocks. Conceivably, therefore, these high-temperature lithotypes have the potential to mute primary atmospheric signals via post-depositional sulphur remobilisation and/or mixing with non-atmospherically processed volcanogenic sulphur[12].

Leaving the atmospheric significance of the diamictite-derived QSI data from the succeeding Diamictite/Greywacke Member as an open question (see Supplementary Section 1.3 below), we note the presence of a dolarenite that features elevated $\Delta^{3X}S$ values and Archean-like $\Delta^{36}S/\Delta^{33}S$ systematics (slope = -1.44). While Warke et al. ascribe these clearly mass-independent QSI systematics to crustal recycling[10], we *stress* that the negative $\Delta^{33}S$ and positive $\Delta^{36}S$ values possessed by this sample strongly conflict with previous computational[13] and empirical[1,2] estimates of the crustal memory effect. Indeed, as we affirm herein, the available evidence suggests that a crustal memory effect would be expected to have positive $\Delta^{33}S$ and negative $\Delta^{36}S$ values during this time interval. Ignoring a potentially unrecognised volcanic overprint, we are thus left with a potential detrital origin for the sulphur within the measured Polisarka dolarenite. Here, while we cannot preclude the counter-effect of shortened transit-time within more energetic sedimentary regimes, the accumulation of detrital sulphide grains against the backdrop of a "major oxygenation event" seems somewhat counterinitiative and requires thorough investigation.

Returning to South Africa, while the ages of the Kaapvaal-housed Koegas Subgroup remain the subject of active debate[14,15,16], the overlying Makganyene diamictite, dated to 2,424 ± 32 Ma, is presumed to be correlative with the diamictite within the uppermost Polisarka Sedimentary Formation[8,10] (Supplementary Fig. 2). This correlation is significant because, besides presenting a more spatially widespread evaluation of atmospheric chemistry, examination of the roughly time-equivalent strata preserved within the Griqualand-West sub-basin reveals a pronounced dichotomy in sulphur isotope systematics. Here, while bulk SF_6 analysis of the Rooinekke Formation captured within two cores returned $\Delta^{33}S$ values around 0‰, SIMS analysis of the same samples revealed a ~7‰ range $\Delta^{33}S$ values, extending down to some of the most pronounced negative values encountered throughout the entirety of Earth's history (Supplementary Fig. 2)[17]. Taken together, these South African QSI systematics can only be explained as photochemical derivatives, which strongly contrasts with the inferences drawn from Fennoscandia. Understanding whether this incongruity is the product of inadequate age control, or issues concerning atmospheric fidelity within the Fennoscandian record remains an open question and, as such, presents a significant obstacle to conclusively associating the first major Snowball Earth event with a major oxygenation episode/event. For now, given the data in hand, the significance and, indeed, the loss of S-MIF within Fennoscandia, remains to be conclusively demonstrated.

1.2.2 Age Equivalence of the Duitschland and Rooihoogte Formations?

The second and potentially third oxygenation episode(s) proposed by Gumsley et al.[8] were inferred from the QSI systematics of the South African Duitschland and Rooihoogte formations (Supplementary Fig. 1). The QSI systematics of both of these units feature a pronounced shift from large $\Delta^{3X}S$ values and Archean-like $\Delta^{36}S/\Delta^{33}S$ slopes to essentially zero $\Delta^{3X}S$ values with mass-dependent $\Delta^{36}S/\Delta^{33}S$ slopes[9,18–20]. Unfortunately, the Duitschland and Rooihoogte formations lack direct age constraints, being constrained, currently, by consistent Re–Os isochron (2,316 ± 7 Ma[21]) and U–Pb zircon (2,310 ± 9 Ma[22]) ages obtained from the overlying lower Timeball Hill Formation and a U–Pb zircon age (2,480 ± 6 Ma[23]) from the unconformably preceding Penge Iron Formation[24] (Supplementary Fig. 1). Using detrital zircon U–Pb derived ages, Schröeder et al. assigned a maximum depositional age of ≤ 2,434 ± 16 Ma for the Duitschland Formation[25]; however, more recent detrital zircon work has revealed age distributions for the Rooihoogte and Duitschland formations that are broadly similar, implying maximum depositional ages of < 2,353 ± 18 and < 2,343 ± 18 Ma, respectively[7,26]. While the veracity of the youngest of these ages has been contested[5], it remains unclear whether these detractions inhibit direct correlation between the Duitschland and Rooihoogte formations, especially given the striking similarity between their zircon age assemblages and distributions[26]. Nevertheless, in the absence of precise age constraints, sedimentological and sequence stratigraphic arguments have been employed to examine the relationship between the Duitschland and Rooihoogte formations[3,5,7,8,24,27], leaving opinions divided. Here, while some authors claim that their equivalence supports a single oxygenation event[10,19], others place the Duitschland Formation below the Rooihoogte Formation, requiring two distinct episodes of oxygenation[3,8,9] (Supplementary Fig. 1). Direct ages from both the Duitschland and Rooihoogte formations are essential to resolve these opposing hypotheses.

1.2.3 Time Elapsed Between the Last Appreciably Negative and Positive $\Delta^{33}S$ Value

To begin, it is useful to reiterate how the ages utilised throughout this study are defined (see Table 1). The minimum and maximum ages for a given sample are defined by the nearest published ages above and below that sample respectively, with a few select ages being extrapolated from sedimentary relationships where they are well defined (Supplementary data file New_MIF_database.xlsx). For example, the max age of the Rooihoogte formation is defined by the maximum age of the sequence boundary that cuts across the Transvaal, rather than the age in the Penge Iron formation[8] (Supplementary Fig. 1). This rather broad approach is the most geochemically 'honest' and practical, but can in certain cases lead to a sample being ascribed an age that is younger or older than what the sample most likely is. As for how this relates to the discussion of the age offset between the last appreciably negative and positive $\Delta^{33}S$ value, the youngest value that is statistically-significantly lower than -0.3‰ is from the Boolgeeda Iron Formation of the Turee Creek group, Australia. However, as discussed above (Supplementary Section 1.1) the upper bounding ages of the Turrey Creek group are contested. If we adhere to the age model of Philippot et al., (2018), and take the strict mean of the nearest older (2454 ± 23 Ma) and nearest younger age (2340 ± 22 Ma) we arrive at a mean age of 2397 Ma. Uncertainties in the upper age aside, the true age of the samples that host the negative $\Delta^{33}S$ values is likely much closer to 2450 Ma as the upper age constraint is located >1.5 Km up-section from these data. A ca. 2454-2420 Ma age for these samples would also be consistent with data from the Griqualand west subbasin, specifically within the. Heinskop/Rooinekke formations of the Ghaap group, South Africa. While the age constraints here are again, contested (Supplementary Section 1.2.1), the pronounced negative $\Delta^{33}S$ values there (some of the most negative in the record) are thought to be older than 2426 Ma, and potentially younger than 2436 Ma[16] (Supplementary Fig. 2).

It is also pertinent to distinguish between the youngest S-MIF under a definitively anoxic atmosphere, and the youngest positive S-MIF writ-large. As discussed in the main text, it seems the Rooihoogte formation represents a fundamental turning point in Earths redox balance, and as such can be considered the last vestige of an "Archean-like" atmosphere. Whereas the data of Poulton et al. in the Timeball Hill formation, seem to be more related to some form of sulphur cycle dynamics under a more oxidized atmospheric backdrop. So, as it relates to the discussion of low-level oxidative weathering of pyrite under an atmosphere capable of producing/preserving S-MIF, using the Rooihoogte formation as the youngest positive S-MIF is more apt, but not "correct" in the strictest sense. The youngest significantly positive value prior to the data in the Timeball Hill formation presented by Poulton et al., 2021, is in the Rooihoogte formation (up to +9‰)[9,19,20], which within best available age constraints have a max age of 2353 ± 18 Ma, min age of 2316 ± 7 Ma and mean age of 2334 Ma (Fig. 1) which is consistent with the ages proposed by Luo et al., (2016) of 2330 Ma based on sedimentation/compaction rate estimates[19,21]. So, depending on the age model

one prescribes to, and whether one takes the strictest definition of sedimentary age constraints, or utilizes some form of sedimentation rate estimates, the spread between the youngest large negative and positive $\Delta^{33}S$ values could be on the order of 60 Myr, or closer to 100 Myr. We tend to lean towards the larger of those two estimates, but the case can be (and has been[10]) made that they were closer in age. As for the Timeball Hill data of Pouton et al., the appreciably positive $\Delta^{33}S$ values they report are predominantly between 2316 and 2256 Ma, but there are several that are decidedly younger than 2256. Therefore, if we use these as the youngest positive S-MIF the spread becomes >200 Myr but this age-range is misleading in the context of pyrite oxidation in the lead up to the GOE.

1.3 The Utility of Glaciogenic Sediments in Reconstructing Atmospheric Oxygen

While a significant proportion of the post-Rooihoogte-aged EBA "reoccurrences" of S-MIF (i.e., samples whose $\Delta^{33}S$ values exceed 0.3‰) were detected within the Timeball Hill Formation, several instances were recognised within the overlying glaciogenic diamicite (i.e., the Rietfontein Member) and its reworked equivalent (i.e., the lower Boshoek Formation)[9]. Here, targeting the matrix, rather than the clasts, Poulton et al. argued that the elevated $\Delta^{33}S$ values (i.e., > 0.3‰) that they observed within these glacially influenced strata captured the primary operation of atmospheric chemistry, recording rises and falls in pO_2 across the threshold necessary for S-MIF production[9]. Sharing common authors, however, Warke et al. reached an opposing conclusion, citing parsimony to dismiss S-MIF preserved within the high energy facies (i.e., diamictites and arenites) of the Fennoscandian Polisarka Formation as products of crustal recycling[10]. Similarly, others have successfully identified S-MIF-bearing detrital pyrites within older glaciogenic facies[28,29], demonstrating that care is needed when interpreting the MSI systematics of these high-energy lithotypes. As affirmed by Poulton and colleagues[9], Izon et al.[20] failed to identify rounded or pitted pyrite grains in several proximal cores that would imply a detrital origin within the older, and presumably non-glacially impacted, portion of the Carletonville sedimentary succession[19]. Izon et al. did, however, identify a mud-clast supported breccia in a proximal core, KEA-4, that was exceedingly difficult to discern in unpolished core samples[20]. Given the widespread nature of this cryptic facies within the Carletonville area[24], these samples provide an intriguing mechanism whereby hydraulic equivalence arguments are rendered invalid, providing an armoured vector for reworked pyrites to avoid erosion and, therefore, detection via typical morphological metrics. Assimilated, given the conflicting viewpoints concerning the origin of S-MIF within glaciogenic rocks, it seems that detailed petrographic and SIMS-based spatially resolved isotope studies are needed to: (i) unequivocally ascribe the non-zero $\Delta^{33}S$ values within the Rietfontein Formation/Boshoek Member as primary atmospheric derivatives; and/or (ii) dismiss the non-zero $\Delta^{33}S$ values of the Fennoscandian Diamictite/Greywacke Member as reworked/detrital signals.

2 Exploring Biases within the MSI Record:

2.1 Perceptual Bias

A conspicuous feature of the updated record that is generally lost in the typical $\Delta^{3X}S$ *vs* time portrayal (e.g., Fig. 1b) is that much of the record is composed of relatively muted $\Delta^{33}S$ values. For instance, 95% of the pre-2.3 Ga $\Delta^{33}S$ dataset features values that fall below 5.04‰ (95th percentile; Supplementary Fig. 3a–b). Importantly, the preponderance of low $\Delta^{33}S$ values is also observed in the SSA database, with 95% of the data falling below 5.84‰ (Supplementary Fig. 3c), thereby demonstrating that this bias cannot simply be ascribed to an overabundance of low-$\Delta^{33}S$ spot analyses and, instead, captures a real feature of the MSI record. As such, it remains exceedingly difficult to ascribe the large $\Delta^{33}S$ non-detrital values seen within the Rooihoogte Formation (up to +8‰) to crustal recycling or post depositional remobilisation[9,19,20] (Fig. 1c, 5b). Here, such an explanation would require that the bulk of the sulphur housed within the Rooihoogte samples was sourced from the most extreme tail of the geological QSI record and, importantly, this hypothetical sulphur pool managed to avoid mixing with any low-$\Delta^{33}S$ sulphur, which clearly constitutes the vast majority of the record[20] (Supplementary Fig. 3-4).

2.2 Lithological Bias

The minor sulphur isotope database is dominated by analyses of marine shales [*sic*] and associated low-energy siliciclastic rocks (LES; Supplementary Table 1). To assess whether the overall dominance of LES samples within the database had an outsized impact on its mean $\Delta^{33}S$ value, we constructed a simple numerical test. Given that the mean of the entire database can be represented as the sum of the weighted means of each lithotype (Supplementary Fig. 4; Supplementary Table 1), we conducted three separate thought experiments, whereby, drawing *only* from samples

deposited prior to 2.3 Ga, we varied the weight (i.e., percentage or fractional abundance) of the LES lithotype by assigning it (i) a lower (< ~20%), (ii) a higher (> ~60%) and (iii) approximately similar weighting albeit with some variability (42.3 ± ~15%). Here, irrespective of the scenario, the remaining weight was randomly assumed by the remaining lithotypes, permitting calculation of the mean $\Delta^{33}S$ value for each simulation. Repeating this operation 1,000 times then yielded distributions whose means were found to be within ±0.2‰ of the true weighted mean (Supplementary Table 2; Supplementary Fig. 5). This first-order agreement indicates that the over-representation of LES rocks within the database does not have a substantial impact on the mean $\Delta^{33}S$ value of the database until geologically unreasonable LES weightings are used. Combining this insensitivity with the lack of correlation between sulphur content and $\Delta^{33}S$ values[13], suggests that the observed lithological bias can be effectively ignored in our exploration of the CME.

2.3 Spatial Bias

The MSI record is dominated by analyses of rocks originating from South Africa and Australia. Regardless of whether the full or SSA database was considered, the Kaapvaal and Pilbara cratons account for 70–75% of the available pre-2.3 Ga $\Delta^{33}S$ data (Fig. 2b; Supplementary Table 3). Such bias is further highlighted by the fact that 46% of the entire database, or 38% of the SSA database, is derived from just 10 formations. Surprisingly, outside of these cratons, there are only six instances where $\Delta^{33}S$ values exceed 5.0‰ and, of those, all but one datapoint are spot analyses from the same stratigraphic interval. Clearly the large $\Delta^{33}S$ values seen in the Kaapvaal and Pilbara cratons are not necessarily representative of the wider record, and their overrepresentation exerts an inordinate weight on the cumulative $\Delta^{33}S$ mean.

3 Point Source Injection of Sulphur *via* Plinian Eruptions:

As discussed in the main text, even under the relatively low sulphate concentrations of the Paleoproterozoic ocean (estimated to be on the order of millimoles/kg[19,29–31]) it is exceedingly unlikely that $\Delta^{33}S$ values similar those seen in modern ice cores would be preserved in the wake of massive Plinian style eruptions[32–34]. A potential workaround to the mass-balance issues that prevent the preservation of such events in the sedimentary record could potentially be found in the model put forward by Gallagher et al. (2017)[35]. Gallagher and colleagues proposed that anomalously large $\Delta^{33}S$ values of the Neoarchean were the result of a massive point-injections of sulphur into back-arc basins by volcanic eruptions, wherein the emitted sulphur aerosols had a positive $\Delta^{33}S$ starting composition resulting from the recycling of S-MIF bearing sediments from a subducting slab. Once put into the atmosphere "*UV photolysis proceeded to fractionate the already positive $\Delta^{33}S$ of the volcanic aerosol to even higher values (+ 9.46 ‰ $\Delta^{33}S$) in the S_8 phase, and lower the sulphate phase towards lower, but still positive values (ca. + 3.5 ‰ $\Delta^{33}S$)*"[35]. So, inherently, this idea requires that the sulphur being emitted by specific volcanic eruptions during the Neoarchean must have had a starting composition somewhere between 3.5 and 9.46‰, with other records requiring less extreme starting compositions. However, it is difficult to square this prerequisite with the average composition of the pre-Neoarchean record, 0.15 and 0.19‰ for the full and spot sample averaged database respectively (Supplementary Table 4.1). Indeed, even the composition of the suggested negatively fractionated sulphate phase (3.5 ‰) is outside of the 97.5th quantile (q97.5 in Supplementary Table 4.1), meaning that more than 97.5 % of the pre-Neoarchean record cannot provide a sufficiently large $\Delta^{33}S$ value to the recycled volcanic sulphur pool to even reach the negatively fractionated endmember of this model. This issue is even more acute when only the Mesoarchean data are considered (Supplementary Table 4.2), which are notably muted compared to the rest of the Archean and Paleoproterozoic (Fig. 1; Supplementary Fig. 3). Even if we take the most unlikely case and apply this analysis to the entire pre-2.3 Ga record, 3.5 ‰ still lies outside of the 85th quantile (Supplementary Table 4.3). It should be noted that all the statistical analyses presented here were performed on the raw/SSA database without any bias correction, which would be expected to further reduce the mean and specific quantile associated $\Delta^{33}S$ values.

There is also the compounding issue that deep sea sediments/oceanic crust, which would presumably be the most voluminous source to arc magnetism, are on average expected to host proportionally larger amounts of the negative $\Delta^{33}S$ endmember[13,36–39] thereby driving the positive skew observed in the continental record. Though we have shown that the positive skew is much diminished, it does still exist in our bias corrected record, reinforcing the need for an accompanying negative sink, most likely in the subducting oceanic crust. In summary, these observations undercut one of the main premises of the Gallagher model, by requiring not only that recycled S-MIF bearing sulphur be the dominant source of sulphur in the erupted aerosols, but also that its $\Delta^{33}S$ composition be sourced from the extreme

tail of the known record, in a reservoir that is not even expected to host a significant amount of that extreme tail[13,36–39]. Even the Timeball Hill data of Poulton et al., showing a maximum $\Delta^{33}S$ of 2.9‰, which would be at least partially diluted by the existing SWSR, still fall victim to the same issues.

4 Sensitivity Test of Bootstrap Sampling Parameters:

To examine the sensitivity of our bootstrap subroutine to the sample size (*n,*) and the number of replications (*m*), we applied the function to the sample-resampled database at t = 2,300 Ma, repeating this operation 10 times to obtain an average. As can be seen in Supplementary Fig. 6a, the spread in the calculated grand means was highest at low *n* and *m* values and *vice versa*. This is most easily visualised via computation of the standard deviations whose spread, and magnitude dramatically decrease as *n* and *m* increase (Supplementary Fig. 6b). That said, the centre of the grand mean distributions stays relatively constant irrespective of the chosen *n*, especially at higher *m* values, aligning with the weighted mean. The benefit of using a bootstrap subroutine, rather than a simple weighted mean, is that it yields an estimate of variability that is not captured by the mean of the database alone. For instance, we can now explore the influence of craton sizes and/or weatherability that could promote regional biases in the weathered sulphur flux. Alternatively, the observed spread in the CME can be viewed as the potential $\Delta^{33}S$ extremes that could dominate the sulphur pool within an isolated/restricted marine basin, allowing for some regional variability in the observed CME. Values of 10 and 20 were used for *n* and *m* respectively in the simulations described in the main text (Figs. 3 and 4).

Supplementary Figures:

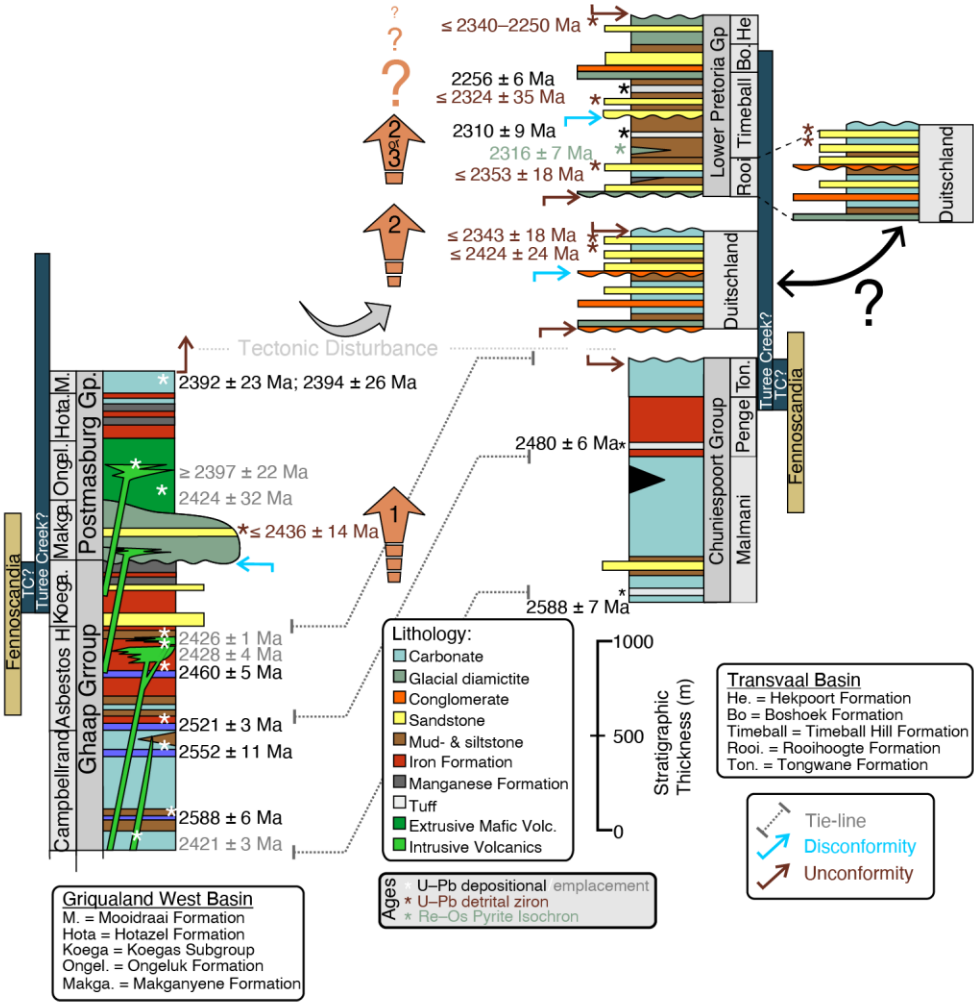

Supplementary Figure 1: A litho- and chrono-stratigraphic synthesis of the Transvaal Supergroup, building on that presented by Gumsley et al.[8] and from Izon and Luo et al.[20]. Orange vertical arrows in the center denote the hypothesised oxygenation episodes discussed in Supplementary Section 1, while the question marks signal the spatial uncertainty highlighted by from Izon and Luo et al.[20]. Solid blue vertical bands illustrate the conflicting interpretations of Philippot et al.[1,7] and Bekker et al.[3,5] concerning the age of the Turee Creek (TC) group. Solid tan vertical bands show the approximate position of the Fennoscandian loss of S-MIF[10] (See Supplementary Fig. 2) and its clear separation from the Duitschland/Rooihoogte from Izon and Luo et al.[20]. The black double-ended arrow shows the contrasting stratigraphic placements of the Duitschland Formation relative to the Rooihoogte Formation. All ages shown are discussed by Gumsley et al.[8], or referenced in Supplementary Section 1.

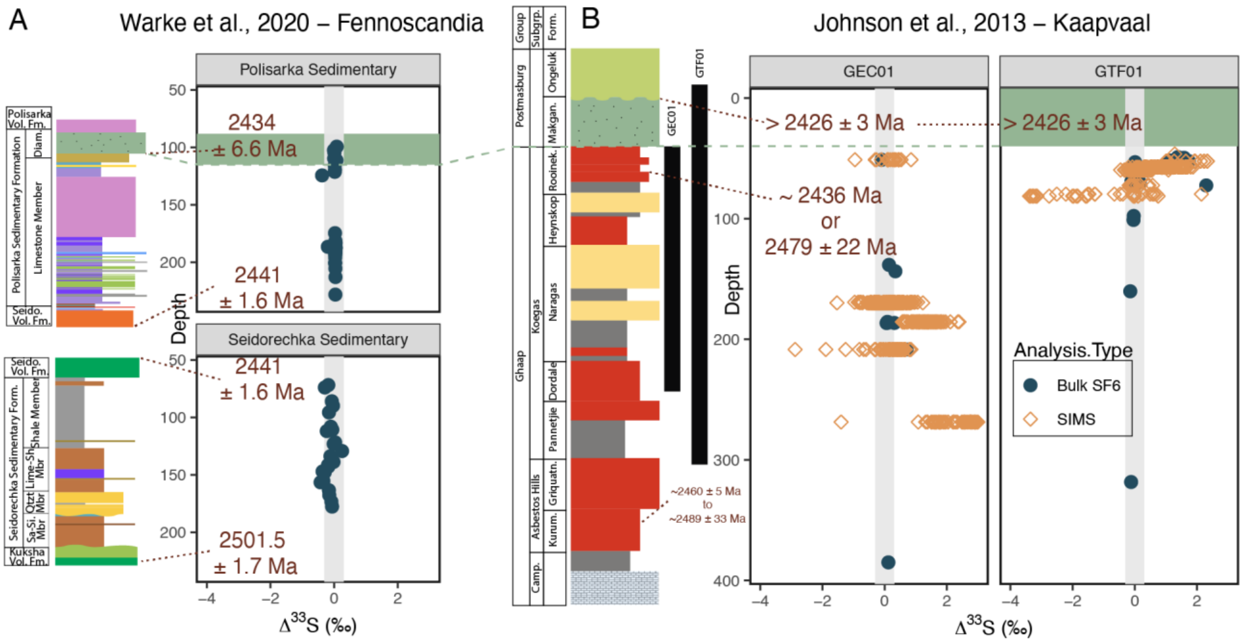

Supplementary Figure 2: Potentially age-equivalent Δ^{33}S data from Fennoscandia (A) and the Griqualand West Basin, Kaapvaal Craton (B). Here, the lithostratigraphy and chronological constraints (red text) follow those provided in the original publications[10,26], as discussed in Supplementary Section 1.2.1. Closed and open data points distinguish between SF_6- and SIMS-derived Δ^{33}S data. The vertical grey bars illustrate a ±0.3‰ Δ^{33}S-envelope centred on 0‰, representing the conservative threshold for S-MIF determination[9]. The horizontal green bar in panel A identifies the Greywacke-Diamictite Member of the Polisarka Sedimentary Formation, while its equivalent in panel B highlights the supposedly equivalent Makganyene diamictite, which is only captured in core GTF01. The chronological constraints pertaining to the Rooinekke Formation are disputed (Supplementary Section 1.2.1).

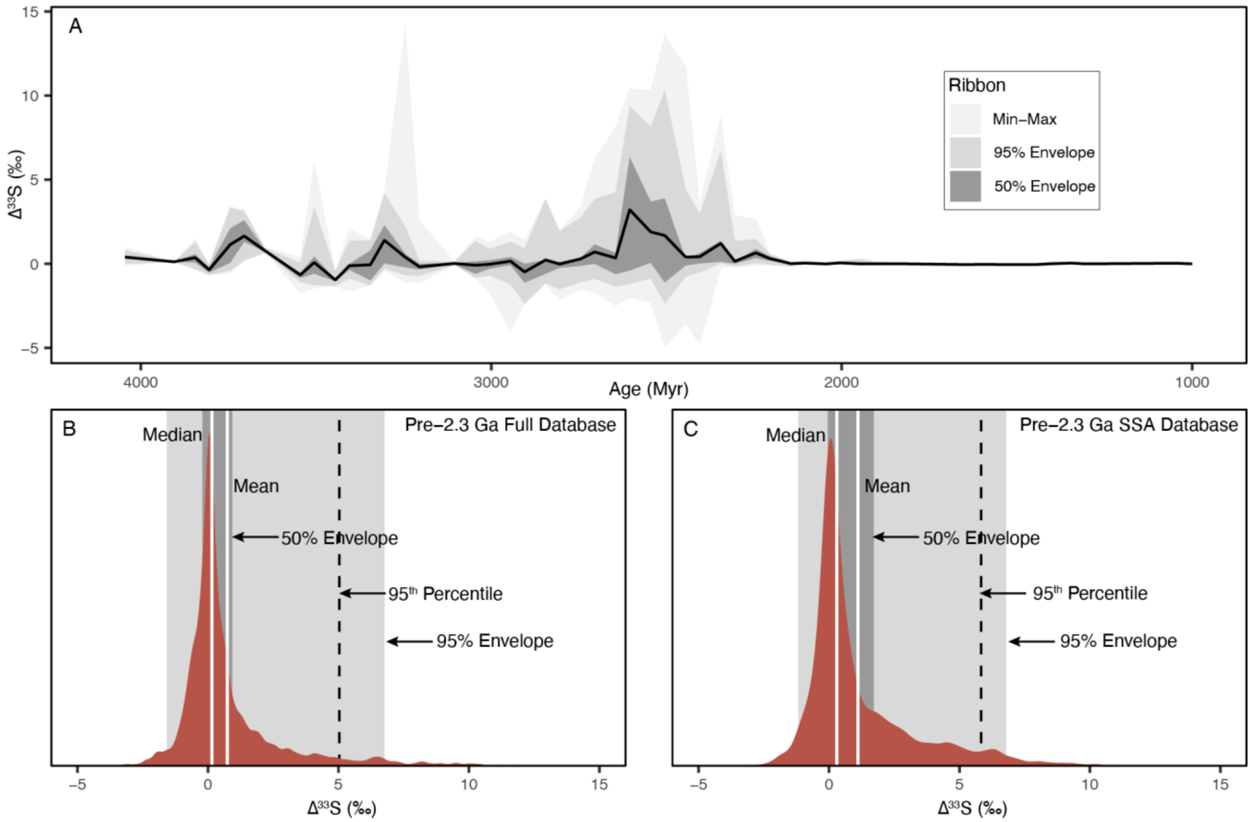

Supplementary Figure 3: Different visualisations of the pre-2.3 Ga $\Delta^{33}S$ dataset. Panel A shows the full database binned in 50-million-year intervals. The black line tracks the bin-specific mean $\Delta^{33}S$ value, while the lightest grey envelope displays the minimum and maximum $\Delta^{33}S$ values captured by each bin. Progressively darker grey envelopes display the spread of the data when only 95% (i.e., those between the 2.5–97.5 quantiles) and 50% (i.e., those between the 25–75 quantiles) of the bin-specific dataset are considered. Here, even in the bins featuring the most extreme $\Delta^{33}S$ values, it is important to note that the maximum mean $\Delta^{33}S$ value is 3.2‰. Panel B illustrates the distribution of the same $\Delta^{33}S$ dataset shown in panel A, while panel C presents an equivalent diagram using the pre-2.3 Ga Spot Sample Averaged (SSA) dataset. In both lower panels, the progressively darker grey envelopes illustrate the spread encompassed by 95% and 50% of the dataset. Vertical white lines show the median and mean of the distribution, while the vertical black dashed line indicates the 95th percentile.

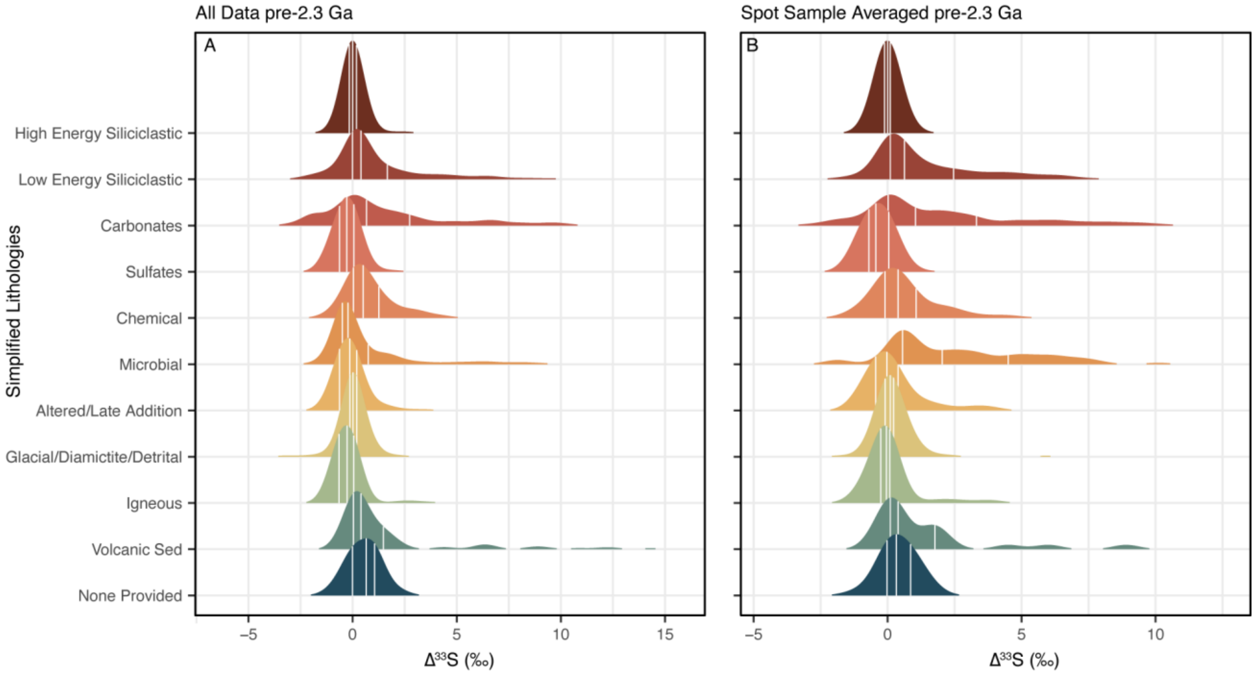

Supplementary Figure 4: Lithology-specific Δ^{33}S distributions. Panel A shows the entire pre-2.3 Ga database, while panel B depicts the SSA database. In both panels, vertical white lines depict the median surrounded by the 25th and 75th quartiles. Note, differing slightly from the text, here Igneous and Volcanic sed. were split in Supplementary Fig. 4 for clarity.

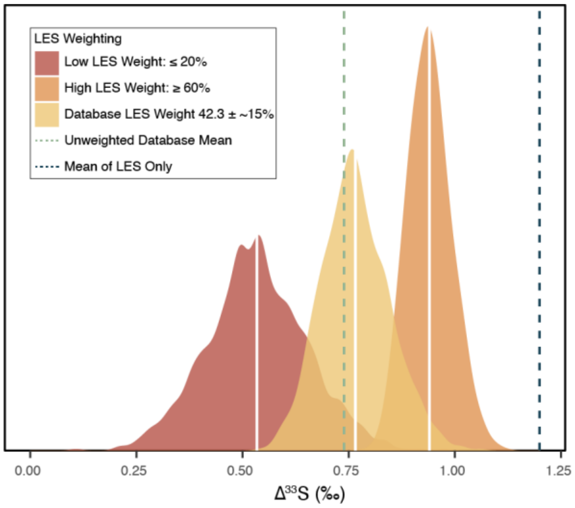

Supplementary Figure 5: Examination of lithological biases. Here, the colour coded distributions show the outcomes of a series of numerical sensitivity tests whereby a low (red), medium (yellow) and high (orange) weighting was prescribed to the low energy siliciclastic (LES) lithotype. Superimposed on the resultant distributions, the vertical white lines indicate the mean of each experimental outcome compared to that of the unweighted mean of the entire pre-2.3 Ga dataset (green dashed line) and its (LES) component (blue dashed line).

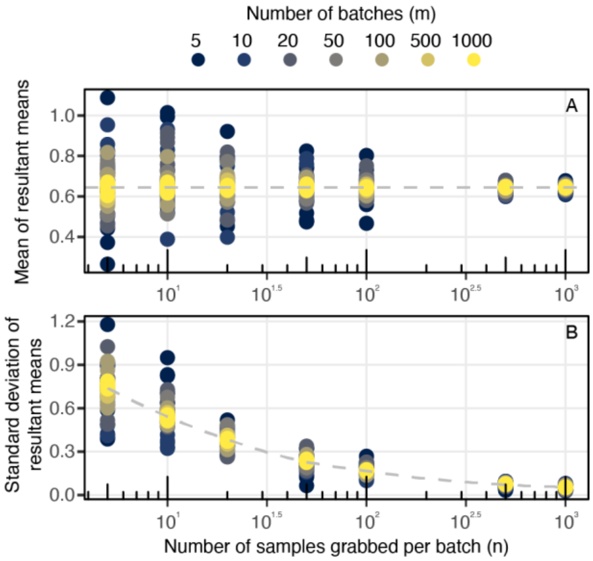

Supplementary Figure 6: Sensitivity analysis of our bootstrap sampling routine conducted at 2.3 Ga. Here, panel A shows the influence of the sample size (*n,* x-axis), and the number of replications (*m*, colour scale), on the $\Delta^{33}S$ estimate (i.e., the grand mean) of the weathered sulphur flux, superimposed on its weighted average. Maintaining a common x-axis and colour scale, Panel B shows the standard deviation of grand means (y-axis) of the data plotted in panel A together with their LOESS fit.

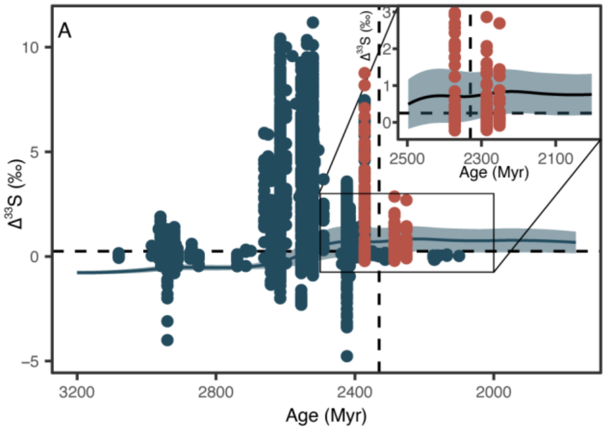

Supplementary Figure 7: Simulations of the crustal memory effect (CME) within a hypothetical hydrographically restricted basin adjacent to the Kaapvaal Craton. (A) Here, the blue line indicates the simulated $\Delta^{33}S$ evolution of the Kaapvaal-specific weathering flux, with its associated envelope capturing the 1σ uncertainty. Superimposed on these, the available Kaapvaal-derived $\Delta^{33}S$ data are plotted in blue, while those from the Timeball Hill formation reported by Poulton et al are distinguished in red[9]. The vertical dashed line marks the GOE *sensu* Luo et al.[19], while the horizontal dashed line represents the globally expressed CME modelled using the CA dataset

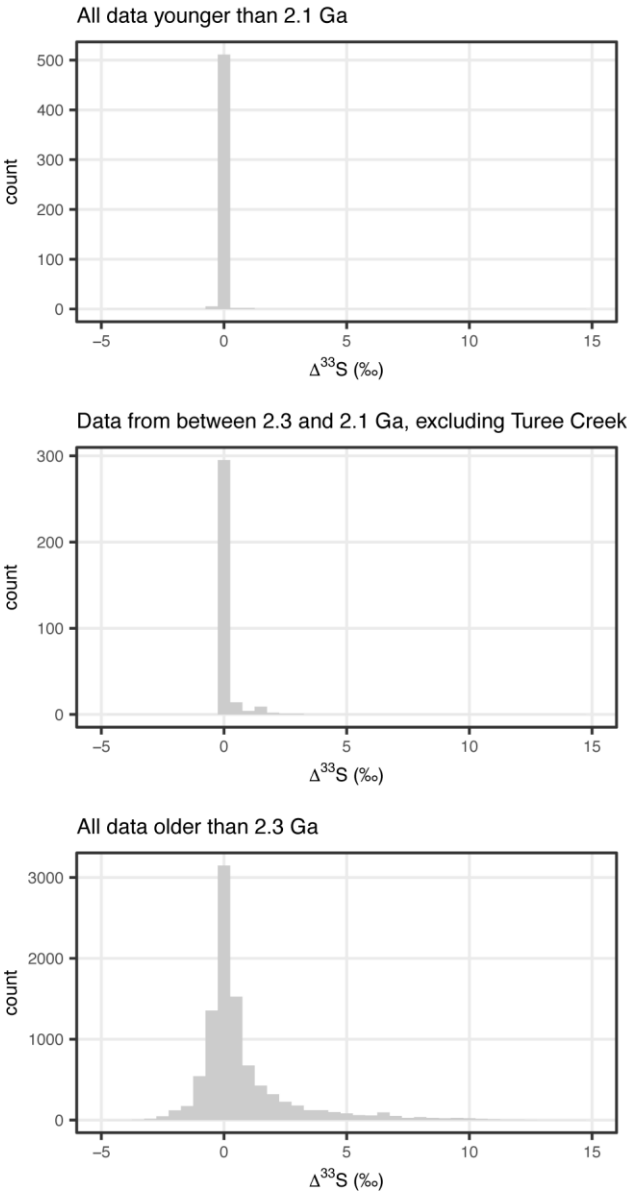

Supplementary Figure 8: Distributions of $\Delta^{33}S$ before during and after the GOE. Top panel contains all data that are younger than 2.1 Ga; Middle panel contains all data from between 2.3 and 2.1 Ga excepting the Turee Creek group; Bottom panel contains all data older than 2.3 Ga

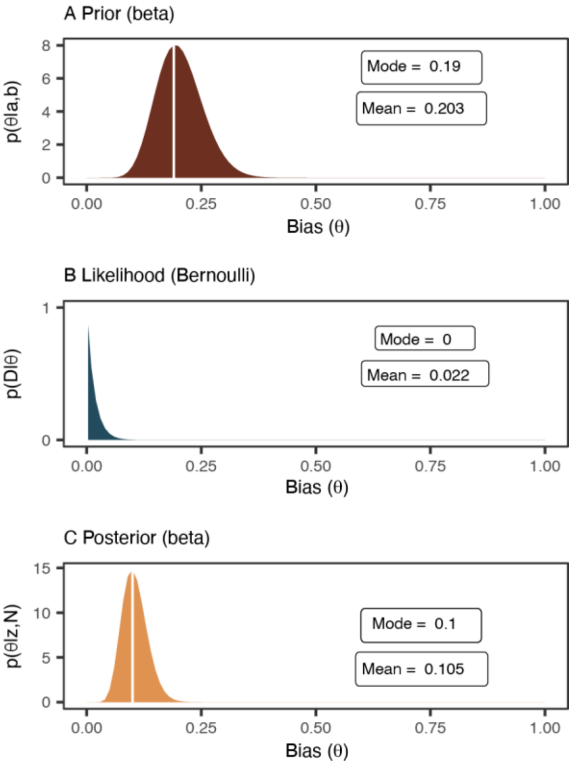

Supplementary Figure 9: Bayesian analysis of the discrepant Carletonville $\Delta^{33}S$ records. Panels a and b give the respective prior (beta) and likelihood (Bernoulli) distributions parameterised using data from cores EBA-2[9] and KEA-4[20]. Their combination then results in the posterior distribution (beta) shown in panel c. Central tendencies are given in each panel, with the mean illustrated as a vertical white line.

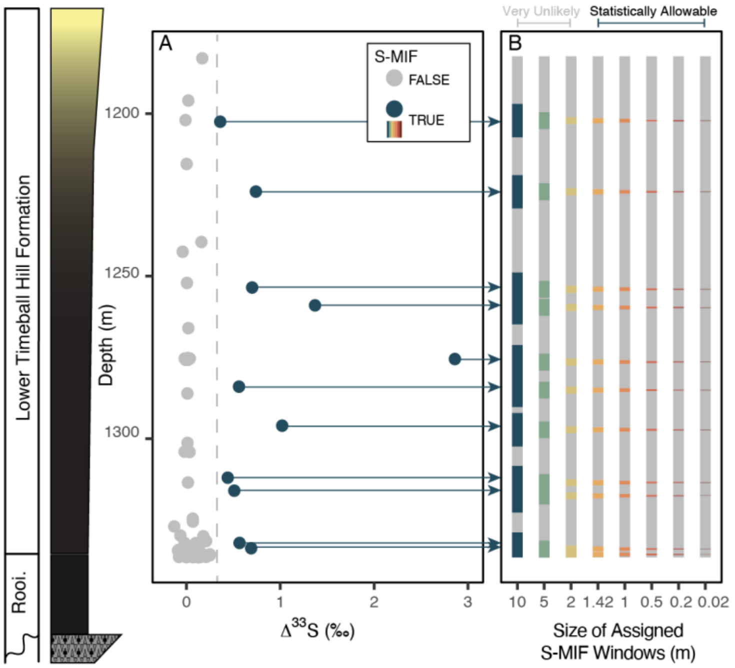

Supplementary Figure 10: Construction of the data-informed synthetic cores used within our statistical experiments. (a) Data points correspond to those reported from the 151.6-m-thick lower Timeball Hill Formation (i.e., beneath the Gatsrand Member) within core EBA-2[9] (Supplementary Table 4). Based on their Δ^{33}S magnitude (Δ^{33}S ≥ 0.3‰, vertical grey dashed line), colour is used to distinguish between samples that possess S-MIF (blue, TRUE) and S-MDF (grey, FALSE). (b) Visualisation of the synthetic cores constructed using the stratigraphic position of S-MIF samples reported by Poulton et al.[9]. Colours relate the assigned stratigraphic thickness of the S-MIF window size (x-axis), corresponding to those shown in Fig. 5. Here, for clarity, the thickness of the 0.02-m-thick S-MIF window size is vertically exaggerated. Rooi. abbreviates Rooihoogte Formation.

Supplementary Tables:

Supplementary Table 1: Simplified lithological breakdown of > 2.2 Ga $\Delta^{33}S$ database.

Simplified Lithotype	Mean $\Delta^{33}S$ (‰)	Proportion of Database (%)
High Energy Siliciclastic	0.06	4.69
Low Energy Siliciclastic	1.05	42.3
Carbonates	1.51	5.36
Sulphates	-0.25	5.96
Chemical	0.78	14.8
Microbial	0.64	4.12
Altered/Late Addition	-0.09	5.28
Glacial/Diamictite/Detrital	0.12	9.61
Igneous	-0.22	6.05
Volcanic Sed	1.56	1.02
None Provided	0.52	0.78

Supplementary Table 2: Outcomes of numerical weighting experiments designed to explore the significance of the analytical bias towards analysis of low-energy siliciclastic (LES) lithotypes. Only samples older than 2.2 Ga were used for these sensitivity tests.

Database Weighting	Mean $\Delta^{33}S$ (‰)
Low LES Weight (X)	0.52
High LES Weight (X)	0.85
LES Weight ± ~15%	0.71
Unweighted Mean	0.67
Mean of LES only	1.05

Supplementary Table 3: Spatially distinguished pre-2.3 Ga average $\Delta^{33}S$ values ranked by their representation within the entire database and its spot sample averaged counterpart (SSA).

Rank	Full Database			Spot Sample Averaged (SSA) Database		
	Craton	Mean $\Delta^{33}S$ (‰)	*n*	Craton	Mean $\Delta^{33}S$ (‰)	*n*
1	Kaapvaal	1.20	3890	Kaapvaal	1.80	1184
2	Pilbara	0.57	3223	Pilbara	0.79	612
3	Yilgarn	0.16	809	Superior	0.58	166
4	Superior	0.68	576	São Francisco	-0.05	144
5	São Francisco	-0.20	503	Yilgarn	0.09	84
6	Dharwar	-0.19	208	Dharwar	0.01	76
7	North Atlantic	1.05	191	Zimbabwe	0.35	56
8	Zimbabwe	0.19	68	Fennoscandia	-0.06	50
9	Fennoscandia	-0.06	50	North Atlantic	1.04	49
10	Amazon	0.66	49	North China	-0.07	34
11	North China	-0.07	34	Amazon	0.37	31
12	Not listed	-0.48	6	Not listed	-0.50	5

Supplementary Table 4.1: Comparison of potential sedimentary sulphur sources to Neoarchean volcanoes. sd = standard deviation; med = median; q## represent quantiles of the distribution (eg q95 is the 95th quantile)

Full pre-Neoarchean database							
mean	*sd*	*med*	*q2.5*	*q25*	*q75*	*q95*	*q97.5*
0.148	1.09	-0.06	-1.16	-0.419	0.4	2.07	2.85
**SSA pre-Neoarchean database**							
mean	*sd*	*med*	*q2.5*	*q25*	*q75*	*q95*	*q97.5*
0.185	0.888	0.0233	-1.11	-0.27	0.45	2.05	2.64

Supplementary Table 4.2:

Full Mesoarchean only database							
mean	*sd*	*med*	*q2.5*	*q25*	*q75*	*q95*	*q97.5*
-0.116	0.68	-0.08	-1.77	-0.366	0.103	0.9	1.26
**SSA Mesoarchean only database**							
mean	*sd*	*med*	*q2.5*	*q25*	*q75*	*q95*	*q97.5*
0.0581	0.647	0	-1.07	-0.2	0.2	1.2	1.3

Supplementary Table 4.3:

Full pre-2.3 Ga database								
mean	*sd*	*med*	*q2.5*	*q25*	*q75*	*q85*	*q95*	*q97.5*
0.739	1.97	0.151	-1.58	-0.21	0.925	1.95	5.03	6.77
**SSA pre-2.3 Ga database**								
mean	*sd*	*med*	*q2.5*	*q25*	*q75*	*q85*	*q95*	*q97.5*
1.11	2.06	0.3	-1.17	-0.044	1.72	3.05	5.82	6.79

Supplementary Table 5: Comparison of $\Delta^{33}S$ systematics between different cored expressions of the lower Timeball Hill Formation. Data from the EBA cores is from Poulton et al.[9], while that from core KEA-4 is from Izon and Luo et al.[20]. Data derived from repeated analysis of the same sample were averaged and, therefore, each sample is only included once. Samples with $\Delta^{33}S \geq 0.3‰$ were classified as S-MIF bearing.

Core (Study)	Interval Length (m)	No. of Samples	Mean Gap Between Samples (m)	Max Gap Between Samples (m)	No. of S-MIF-bearing Samples
EBA-1 (Poulton et al., 2021)	168.5	28	6.24	15	2
EBA-2 (Poulton et al., 2021)	151.6	36	4.33	21.5	11
KEA-4 (Izon et al., 2022)	158.3	60	2.64	9.57	0

Supplementary Table 6: Results of our bootstrap sampling experiments conducted using synthetic cores with variable stratigraphic S-MIF distributions. Centred on S-MIF-bearing samples[9], the S-MIF window denotes the prescribed thickness of S-MIF within a given synthetic core (Supplementary Fig. 10). The resultant means and standard deviations are rounded to the nearest whole number (Fig. 5).

S-MIF Window (m)	Mean Number of S-MIF-bearing samples	Standard Deviation (σ)	Percentage of Batches with Zero S-MIF-bearing Samples (%)
0.02	0	0	82
0.20	1	1	39
0.50	2	1	10
1.00	4	2	1
1.42	6	2	0.2
2.00	9	3	0
5.00	19	4	0
10.0	34	4	0

Supplementary Table 6: Lithological simplification used herein. The database synthesises data from 84 papers[1,2,29,37–45,9,46–51,51–54,10,55–64,11,65–74,17,75–84,18,85–94,19,95–104,20,105–107,28]. From these, we take the original lithological descriptors and reassign them to one of the following broad and simplified lithotypes (simp.lith). Idiosyncrasies/typos present in the lithologies column derive from the original publication.

simp.lith	Lithologies
High Energy Siliciclastic	Quartzite, Sandstone, Metasandstone, Argillaceous sandstone, Conglomerate, Coarse Sandstone to Gravel, Quartz pebble conglomerate with 10 vol.% rounded coarse sand, Ooidal sandstone, Quartz Rock, Dolomitic Quartzite, Koolbye quartzite, Conglomerate (Marine), Sandstone, Crossbedded, Dolaenite, Calcarenite, Pyrite-Dolomite-Albite-Sericite Altered Conglomerate
Low Energy Siliciclastic	Black Shale, Shale, Muddy Dolostone, Siliclastic Mudstone, Mudstone-Boundstone Cycles, Calcareous Mudstone, Mudstone, Shaley-Limestone, Magnetic Shale/Mudstone, Non-magnetic shale/mudstone, Carbonaceous Shale/mudstone, Sandstone-Shale Interbeds, Carbonaceous Shale/mudstone-Red, Ankerite bearing-Magnetic Shale/Mudstone, Ankerite bearing-Non-magnetic shale/mudstone, Magnetic Shale/Mudstone/Banded Iron Formation, Shale, Carbonaceous, Siltstone, Black Shale to Siltstone, Biotite-Quartz Schist, Carbonaceous Schist, siltstone, Quartz-garnet-biotite schist, Silstone, Argillite, Metapelite, Sandy Mudstone, Fe-carbonate altered siltstone, Fe-carbonate altered siltston, Silicified siltstone, Medium grey to dark grey turbidic shale, Black carbonaceous shale, Dark to medium grey siltstone, Black carbonaceous siltstone, Dark grey to black interbedded siltstone, Organic Rich Marl, Grey Carbonate, Marl couplet, Black Shale, marl couplets, Carbonated Siltstone, Quartz-Biotite Schists: Finegrained lens, Quartz-Biotite Schists: Clast 1, Quartz-Biotite Schists: Matrix, Quartz-Biotite Schists: Clast 2, Quartz-Biotite Schists: Clast 3, Quartz-Biotite Schists: Clast 4, Quartz-Biotite Schists: Clast 5, Quartz-Biotite Schists: Clast 6, Sedimentary Argillite, Proximal mudstone, Distal shale, Garnet-Biotite Schist (metapelite), Micaschist, Shale, finely laminated, Laminated Black/Grey Shale, Grey Shale, poorly laminated, Grey shale, Sandstone, Grey Shale, Phyllitic Quartzite, Phyllite, Quartzite Schist, Mica Schist, Two-mica Schist, Very fine grained banded dolomitic mudstones, Green mudstone, Mudstone siltstone, Grey mudstone, Laminated mudstone-siltstone, Laminated mudstone-carbonate, Mudtone, Laminated siltstone-mudstone, Laminated carb-mudstone, Mudstone-pyrite layers, Laminated mudstone-carb, Mylonitic Green Schist, Homogeneous mudstone, Laminated mudstone, Black Shale, Carbonate, Siltstone, Carbonate, Black Shale, Siltstone, Siltstone-shale, Limestone-shale, Shale-sandstone, Cherty Shale
Carbonates	Carbonate, Grainy Dolostone, Dolomite, Ferruginous Limestone, Limestone, Carbonate Laminite, Seafloor Aragonite Crystal fan, now Dolomite, Wackestone, Ferruginous Dolomite, Limestone/Dolomite, Limestone, Dolostone, Carbonate, Ooids, Bedded carbonate, Oncolitic dolomite, Fine grained carbonate, Gray carbonate, Clastic Carbonate, Siliceous layer in dolostone, Dolostone (Siliceous layer), Fe-oxide carbonate, dolomite, Greywacke, Carbonate, Laminated
Sulphates	Barite, Silicified Barite, Gypsum, Massive barite, Terrestrial Sulfates, Evaporite, Anhydrite, Pyr. in barite, barite, Bedded Barite

Chemical	Banded Iron Formation, Chert, Black Chert, Iron Formation, Siderite bearing-Iron Formation, Chertified, Black chert, Microscopic sulfide, Macroscopic sulfide, laminated, Macroscopic Sulfide, fine-grained carbonate BIF, Massive Sulfide, Chert from carbonate member, Dolomitic black chert, Barite, Barite-Chert, Interbedded chert and Fe-carbonates, Semi-massive sulfide, Massive sulfide, Siderite-quartz-magnetite banded iron formation (BIF), Jasper-magnetite-chert BIF, Siderite facies iron formation, Macrobarite, Massive sulfide, Layered Magnetite-Quartzite Type Iron Ore Containing Chlorite, Striation Iron Ore With Pyrite, Iron Ore With Large Pyrite Grains, Muscovite Magnetite-Quartzite Type Iron Ore, Muscovite Magnetite-Quartzite Type Iron Ore Containing Pyrite, Iron Ore With Layered Pyrite Grains, Magnetite-Quartzite Type Iron Ore, Iron Ore With Pyrite, Chemical, Banded Iron Formation, Siderite, Chert pebble (K.M. Kere conglomerate), BIF, Black chert pebble (Talya conglomerate), Black chert with dolostone, Banded Iron Formation, Deep Marine/Chemical, Banded Iron Formation/Metaquartzite, Metachert, layered chert, Jasper layer, Sed BC-Core, Sed BC-rim, Sed BC, Silicate grey layered BIF, Silicate grey laminated BIF, Jasper, BIF-cherty, White silicate laminated BIF, Cherty BIF, Laminated BIF, Fe-oxyde carbonate, chert congl., chert, brecc. chert, Algoma-type Banded Iron Formation., Massive Sulfide - Pyritic Filaments, Cherty Dolostone, Carbonate-Chert Breccia
Microbial	Deep Subtidal Microbialite, Microbialite, Draping Calcite, Stromatolitic carbonate, Microbialite, Dolostone, Microbialite, Stromatolite, Stromatolitic Limestone, Silicified Microbial Mat, Stromatolites, Stromatolite, Within barite, Dolomite-Chert Stromatolite, Grey stromatolitic carbonate, Stromatolitic Dolomite, Stromatolitic Chert, Carbonate-microbialite, Black Shale, Stromatolite Limestone, Stromatolite, Dolostone
Altered/Late Addition	Ore body, Massive Ore in BIF, Hydrothermally remobilized from Archean Greenstones, Massive Ore, Hydrothermal, granite related, colloform pyrite, dacite-vein, Disseminated Ore Body, Felsic Hoted Colloform Pyrite, Felsic Hosted Lated Hydrothermal Pyrite, Massive Sulfide Lenses, ore body, Stringer Veins, Chert Vein, Quartz-carbonate-barite-pyrite-sericite alteration, Quartz-carbonate-pyrite-chlorite-sericite alteration, Carbonate vein, Quartz vein in basement granitoid, Quartz-Biotite Schists: Crosscutting vein, Black chert vein, Carbonaceous-schist with pyrite in matrix and quartz veins., Vein Barite, Silica vein, Quartz-Carbonate Vein, Quartz-Albite-Carbonate-Pyrite Vein, Quartz-Pyrite Vein, Quartz Vein, Albite-Dolomite-Biotite-Pyrite Alteration Zones
Glacial/ Diamictite or Detrital	Tillite, Diamictite, Diamictite-Magnetic Shale/Mudstone, Sandstone, Sandstone-Shale Interbeds, Carbonaceous Shale/mudstone, L.Diamictite, Gravity Deposit, Quartz/chert pebble/cobble conglomerate with 40 vol.% rounded pyrite pebbles in a granular quartz sand matrix, Quartz pebble conglomerate with 10 vol.% rounded coarse sand, Fine-grained pyrite pebble conglomerate with 5 vol.% of quartz granules, Quartz pebble conglomerate with 50 vol.% rounded pyrite pebbles, Fine-grained basalt pebble conglomerate with rounded pyrite pebbles (detrital), Quartz pebble conglomerate with 5 vol.% rounded sand, Ooidal sandstone, Argillite, Diamictite-mudstone, Diamictite-sandstone, Diamictite mudstone, Conglomerate, Conglomerate (Marine), Sandstone, Crossbedded, Shale, Glacial, Sandstone, Glacial, Mudstone, Glacial with Dropstones
Igneous or Volcanic Sed	Tuff, Silicified Metabasalt, Barite, Silicified Metabasalt, Volcanic sandstone, Quartz-pyroxene, Interpillow Basalt, Gneiss, Silicified volcanic, Silicified volcanoclastic, Hornblende Magnetite-Quartzite Type Iron Ore Containing Pyrite, Hornblende Magnetite-Quartzite Type Iron Ore, Magnetite Hornblende Quartzite Type Iron Ore, Layered Hornblende Magnetite-Quartzite Type Iron Ore, Hornblende Containing Pyrite, Hornblende Muscovite Magnetite-Quartzite Type Iron Ore, Hydrothermally altered basalt, Volcaniclastic sediment, Quartz vein in metabasalt, Metabasalt, Ameralik dykes, gneiss, Gabbro, felsic ash, felsic volcanics, spherule bed S3, Komatiitic basalt, Inner portion of a barren massive pyrite lens hosted by chloritized metabasalt., Outer portion of a barren semi-massive pyrite lens hosted by chloritized basalt., Pillowed metabasalt., Volcanoclastic, Carbonate, Dolerite, Basalt, Quartz-Pyroxene, Ultramafic Boudin

Supplementary References

1. Philippot, P. *et al.* Globally asynchronous sulphur isotope signals require re-definition of the Great Oxidation Event. *Nat. Commun.* **9**, 2245 (2018).
2. Killingsworth, B. A. *et al.* Constraining the rise of oxygen with oxygen isotopes. *Nat. Commun.* **10**, 1–10 (2019).
3. Bekker, A., Krapež, B. & Karhu, J. A. Correlation of the stratigraphic cover of the Pilbara and Kaapvaal cratons recording the lead up to Paleoproterozoic Icehouse and the GOE. *Earth-Science Rev.* **211**, 103389 (2020).
4. Caquineau, T., Paquette, J.-L. & Philippot, P. U-Pb detrital zircon geochronology of the Turee Creek Group, Hamersley Basin, Western Australia: Timing and correlation of the Paleoproterozoic glaciations. *Precambrian Res.* **307**, 34–50 (2018).
5. Bekker, A., Krapež, B., Karhu, J. A. & Chamberlain, K. Reply to comment on “Bekker, A., Krapež, B., Karhu, J.A., 2020. Correlation of the stratigraphic cover of the Pilbara and Kaapvaal cratons recording the lead up to Paleoproterozoic Icehouse and the GOE. Earth-Science Reviews, 211, 103,389” by Pascal Phili. *Earth-Science Rev.* **218**, 103607 (2021).
6. Krapež, B., Müller, S. G., Fletcher, I. R. & Rasmussen, B. A tale of two basins? Stratigraphy and detrital zircon provenance of the Palaeoproterozoic Turee Creek and Horseshoe basins of Western Australia. *Precambrian Res.* **294**, 67–90 (2017).

7. Philippot, P. *et al.* Comment on 'Correlation of the stratigraphic cover of the Pilbara and Kaapvaal cratons recording the lead up to Paleoproterozoic Icehouse and the GOE' by Andrey Bekker, Bryan Krapež, and Juha A. Karhu, 2020, Earth Science Reviews, https://doi.org/10.1016/. *Earth-Science Rev.* **218**, 103594 (2021).
8. Gumsley, A. P. *et al.* Timing and tempo of the Great Oxidation Event. *Proc. Natl. Acad. Sci.* **114**, 1811–1816 (2017).
9. Poulton, S. W. *et al.* A 200-million-year delay in permanent atmospheric oxygenation. *Nature* **592**, 232–236 (2021).
10. Warke, M. R. *et al.* The Great Oxidation Event preceded a Paleoproterozoic "snowball Earth". *Proc. Natl. Acad. Sci.* **117**, 13314–13320 (2020).
11. Ono, S., Wing, B., Johnston, D., Farquhar, J. & Rumble, D. Mass-dependent fractionation of quadruple stable sulfur isotope system as a new tracer of sulfur biogeochemical cycles. *Geochim. Cosmochim. Acta* **70**, 2238–2252 (2006).
12. Hofmann, A. *et al.* Comparing orthomagmatic and hydrothermal mineralization models for komatiite-hosted nickel deposits in Zimbabwe using multiple-sulfur, iron, and nickel isotope data. *Miner. Depos.* **49**, 75–100 (2014).
13. Reinhard, C. T., Planavsky, N. J. & Lyons, T. W. Long-term sedimentary recycling of rare sulphur isotope anomalies. *Nature* **497**, 100–103 (2013).
14. Schier, K., Bau, M., Münker, C., Beukes, N. & Viehmann, S. Trace element and Nd isotope composition of shallow seawater prior to the Great Oxidation Event: Evidence from stromatolitic bioherms in the Paleoproterozoic Rooinekke and Nelani Formations, South Africa. *Precambrian Res.* **315**, 92–102 (2018).
15. Kendall, B., van Acken, D. & Creaser, R. A. Depositional age of the early Paleoproterozoic Klipputs Member, Nelani Formation (Ghaap Group, Transvaal Supergroup, South Africa) and implications for low-level Re-Os geochronology and Paleoproterozoic global correlations. *Precambrian Res.* **237**, 1–12 (2013).
16. Warke, M. R., Strauss, H. & Schröder, S. Positive cerium anomalies imply pre-GOE redox stratification and manganese oxidation in Paleoproterozoic shallow marine environments. *Precambrian Res.* **344**, 105767 (2020).
17. Johnson, J. E. *et al.* Manganese-oxidizing photosynthesis before the rise of cyanobacteria. *Proc. Natl. Acad. Sci. U. S. A.* **110**, 11238–11243 (2013).
18. Guo, Q. *et al.* Reconstructing Earth's surface oxidation across the Archean-Proterozoic transition. *Geology* **37**, 399–402 (2009).
19. Luo, G. *et al.* Rapid oxygenation of Earth's atmosphere 2.33 billion years ago. *Sci. Adv.* **2**, e1600134 (2016).
20. Izon, G. *et al.* Bulk and grain-scale minor sulfur isotope data reveal complexities in the dynamics of Earth's oxygenation. *Proc. Natl. Acad. Sci.* **119**, (2022).
21. Hannah, J. L., Bekker, A., Stein, H. J., Markey, R. J. & Holland, H. D. Primitive Os and 2316 Ma age for marine shale: Implications for Paleoproterozoic glacial events and the rise of atmospheric oxygen. *Earth Planet. Sci. Lett.* **225**, 43–52 (2004).
22. Rasmussen, B., Bekker, A. & Fletcher, I. R. Correlation of Paleoproterozoic glaciations based on U-Pb zircon ages for tuff beds in the Transvaal and Huronian Supergroups. *Earth Planet. Sci. Lett.* **382**, 173–180 (2013).
23. Nelson, D. R., Trendall, A. F. & Altermann, W. Chronological correlations between the Pilbara and Kaapvaal cratons. *Precambrian Res.* **97**, 165–189 (1999).
24. Coetzee, L. L. Genetic stratigraphy of the Paleoproterozoic Pretoria group in the Western Transvaal. *Dissertation* (RAND AFRIKAANS UNIVERSITY, 2001).
25. Schröder, S., Beukes, N. J. & Armstrong, R. A. Detrital zircon constraints on the

tectonostratigraphy of the Paleoproterozoic Pretoria Group, South Africa. *Precambrian Res.* **278**, 362–393 (2016).
26. Zeh, A., Wilson, A. H. & Gerdes, A. Zircon U-Pb-Hf isotope systematics of Transvaal Supergroup – Constraints for the geodynamic evolution of the Kaapvaal Craton and its hinterland between 2.65 and 2.06 Ga. *Precambrian Res.* **345**, 105760 (2020).
27. Warke, M. R. & Schröder, S. Synsedimentary fault control on the deposition of the Duitschland Formation (South Africa): Implications for depositional settings, Paleoproterozoic stratigraphic correlations, and the GOE. *Precambrian Res.* **310**, 348–364 (2018).
28. Guy, B. M. *et al.* A multiple sulfur and organic carbon isotope record from non-conglomeratic sedimentary rocks of the Mesoarchean Witwatersrand Supergroup, South Africa. *Precambrian Res.* **216**–**219**, 208–231 (2012).
29. Williford, K. H., Van Kranendonk, M. J., Ushikubo, T., Kozdon, R. & Valley, J. W. Constraining atmospheric oxygen and seawater sulfate concentrations during Paleoproterozoic glaciation: In situ sulfur three-isotope microanalysis of pyrite from the Turee Creek Group, Western Australia. *Geochim. Cosmochim. Acta* **75**, 5686–5705 (2011).
30. Canfield, D. E. & Farquhar, J. Animal evolution, bioturbation, and the sulfate concentration of the oceans. *Proc. Natl. Acad. Sci.* **106**, 8123–8127 (2009).
31. Planavsky, N. J., Bekker, A., Hofmann, A., Owens, J. D. & Lyons, T. W. Sulfur record of rising and falling marine oxygen and sulfate levels during the Lomagundi event. *Proc. Natl. Acad. Sci. U. S. A.* **109**, 18300–18305 (2012).
32. Burke, A. *et al.* Stratospheric eruptions from tropical and extra-tropical volcanoes constrained using high-resolution sulfur isotopes in ice cores. *Earth Planet. Sci. Lett.* **521**, 113–119 (2019).
33. Baroni, M., Thiemens, M. H., Delmas, R. J. & Savarino, J. Mass-Independent Sulfur Isotopic Compositions in Stratospheric Volcanic Eruptions. *Science (80-.).* **315**, 84–87 (2007).
34. Savarino, J. UV induced mass-independent sulfur isotope fractionation in stratospheric volcanic sulfate. *Geophys. Res. Lett.* **30**, 2131 (2003).
35. Gallagher, M., Whitehouse, M. J. & Kamber, B. S. The Neoarchaean surficial sulphur cycle: An alternative hypothesis based on analogies with 20th-century atmospheric lead. *Geobiology* **15**, 385–400 (2017).
36. Farquhar, J. & Wing, B. A. Multiple sulfur isotopes and the evolution of the atmosphere. *Earth Planet. Sci. Lett.* **213**, 1–13 (2003).
37. Farquhar, J. *et al.* Pathways for Neoarchean pyrite formation constrained by mass-independent sulfur isotopes. *Proc. Natl. Acad. Sci. U. S. A.* **110**, 17638–17643 (2013).
38. Jamieson, J. W., Wing, B. A., Hannington, M. D. & Farquhar, J. Evaluating isotopic equilibrium among sulfide mineral pairs in Archean ore deposits: case study from the Kidd Creek VMS deposit, Ontario, Canada. *Econ. Geol.* **101**, 1055–1061 (2006).
39. Ono, S. *et al.* New insights into Archean sulfur cycle from mass-independent sulfur isotope records from the Hamersley Basin, Australia. *Earth Planet. Sci. Lett.* **213**, 15–30 (2003).
40. Bao, H., Rumble, D. & Lowe, D. R. The five stable isotope compositions of Fig Tree barites: Implications on sulfur cycle in ca. 3.2 Ga oceans. *Geochim. Cosmochim. Acta* **71**, 4868–4879 (2007).
41. Baumgartner, R. J. *et al.* Sulfidization of 3.48 billion-year-old stromatolites of the Dresser Formation, Pilbara Craton: Constraints from in-situ sulfur isotope analysis of pyrite. *Chem. Geol.* **538**, 119488 (2020).
42. Bekker, A. *et al.* Dating the rise of atmospheric oxygen. *Nature* **427**, 117–120 (2004).
43. Bontognali, T. R. R. *et al.* Sulfur isotopes of organic matter preserved in 3.45-billion-year-old stromatolites reveal microbial metabolism. *Proc. Natl. Acad. Sci. U. S. A.* **109**, 15146–15151 (2012).

44. Bosco-Santos, A. *et al.* Neoarchean atmospheric chemistry and the preservation of S-MIF in sediments from the São Francisco Craton. *Geosci. Front.* 101250 (2021). doi:10.1016/j.gsf.2021.101250

45. Bühn, B., Santos, R. V., Dardenne, M. A. & de Oliveira, C. G. Mass-dependent and mass-independent sulfur isotope fractionation (δ 34S and δ 33S) from Brazilian Archean and Proterozoic sulfide deposits by laser ablation multi-collector ICP-MS. *Chem. Geol.* **312**–**313**, 163–176 (2012).

46. Caruso, S. *et al.* The fluid evolution of the Nimbus Ag-Zn-(Au) deposit: An interplay between mantle plume and microbial activity. *Precambrian Res.* **317**, 211–229 (2018).

47. Caruso, S., Fiorentini, M. L., Barnes, S. J., LaFlamme, C. K. & Martin, L. A. J. Microchemical and sulfur isotope constraints on the magmatic and hydrothermal evolution of the Black Swan Succession, Western Australia. *Miner. Depos.* **55**, 535–553 (2020).

48. Cates, N. L. & Mojzsis, S. J. Chemical and isotopic evidence for widespread Eoarchean metasedimentary enclaves in southern West Greenland. *Geochim. Cosmochim. Acta* **70**, 4229–4257 (2006).

49. Chen, M. *et al.* Multiple sulfur isotope analyses support a magmatic model for the volcanogenic massive sulfide deposits of the Teutonic Bore volcanic Complex, Yilgarn Craton, Western Australia. *Econ. Geol.* **110**, 1411–1423 (2015).

50. Crockford, P. W. *et al.* Claypool continued: Extending the isotopic record of sedimentary sulfate. *Chem. Geol.* **513**, 200–225 (2019).

51. Hodgskiss, M. S. W., Crockford, P. W., Peng, Y., Wing, B. A. & Horner, T. J. A productivity collapse to end Earth's Great Oxidation. *Proc. Natl. Acad. Sci. U. S. A.* **116**, 17207–17212 (2019).

52. Cui, H. *et al.* Searching for the Great Oxidation Event in North America: A Reappraisal of the Huronian Supergroup by SIMS Sulfur Four-Isotope Analysis. *Astrobiology* **18**, 519–538 (2018).

53. Domagal-Goldman, S. D., Kasting, J. F., Johnston, D. T. & Farquhar, J. Organic haze, glaciations and multiple sulfur isotopes in the Mid-Archean Era. *Earth Planet. Sci. Lett.* **269**, 29–40 (2008).

54. Fabre, S. *et al.* Iron and sulphur isotopes from the Carajás mining province (Pará, Brazil): Implications for the oxidation of the ocean and the atmosphere across the Archaean-Proterozoic transition. *Chem. Geol.* **289**, 124–139 (2011).

55. Farquhar, J. & Wing, B. A. Multiple sulfur isotopes and the evolution of the atmosphere. *Earth Planet. Sci. Lett.* **213**, 1–13 (2003).

56. Farquhar, J. *et al.* Isotopic evidence for Mesoarchaean anoxia and changing atmospheric sulphur chemistry. *Nature* **449**, 706–709 (2007).

57. Farquhar, J., Bao, H. & Thiemens, M. Atmospheric influence of Earth's earliest sulfur cycle. *Science (80-.).* **289**, 756–758 (2000).

58. Galić, A. *et al.* Pyrite in a sulfate-poor Paleoarchean basin was derived predominantly from elemental sulfur: Evidence from 3.2 Ga sediments in the Barberton Greenstone Belt, Kaapvaal Craton. *Chem. Geol.* **449**, 135–146 (2017).

59. Golding, S. D. & Glikson, M. *Earliest life on earth: Habitats, environments and methods of detection*. *Earliest Life on Earth: Habitats, Environments and Methods of Detection* (2011). doi:10.1007/978-90-481-8794-2

60. Gregory, D. D. *et al.* Trace element content of pyrite from the Kapai slate, St. Ives gold district, Western Australia. *Econ. Geol.* **111**, 1297–1320 (2016).

61. Gregory, D. D. *et al.* The chemical conditions of the late Archean Hamersley basin inferred from whole rock and pyrite geochemistry with δ33S and δ34S isotope analyses. *Geochim. Cosmochim. Acta* **149**, 223–250 (2015).

62. Hauri, E. H., Papineau, D., Wang, J. & Hillion, F. High-precision analysis of multiple sulfur isotopes using NanoSIMS. *Chem. Geol.* **420**, 148–161 (2016).

63. Hofmann, A., Bekker, A., Rouxel, O., Rumble, D. & Master, S. Multiple sulphur and iron isotope composition of detrital pyrite in Archaean sedimentary rocks: A new tool for provenance analysis. *Earth Planet. Sci. Lett.* **286**, 436–445 (2009).
64. Hou, K. J., Li, Y. H. & Wan, D. F. Constraints on the Archean atmospheric oxygen and sulfur cycle from mass-independent sulfur records from Anshan-Benxi BIFs, Liaoning Province, China. *Sci. China, Ser. D Earth Sci.* **50**, 1471–1478 (2007).
65. Izon, G. *et al.* Multiple oscillations in Neoarchaean atmospheric chemistry. *Earth Planet. Sci. Lett.* **431**, 264–273 (2015).
66. Izon, G. *et al.* Biological regulation of atmospheric chemistry en route to planetary oxygenation. *Proc. Natl. Acad. Sci. U. S. A.* **114**, E2571–E2579 (2017).
67. Johnston, D. T. *et al.* Geochemistry: Active microbial sulfur disproportionation in the mesoproterozoic. *Science (80-.).* **310**, 1477–1479 (2005).
68. Johnston, D. T. *et al.* Evolution of the oceanic sulfur cycle at the end of the Paleoproterozoic. *Geochim. Cosmochim. Acta* **70**, 5723–5739 (2006).
69. Johnston, D. T. *et al.* Sulfur isotope biogeochemistry of the Proterozoic McArthur Basin. *Geochim. Cosmochim. Acta* **72**, 4278–4290 (2008).
70. Kamber, B. S. & Whitehouse, M. J. Micro-scale sulphur isotope evidence for sulphur cycling in the late Archean shallow ocean. *Geobiology* **5**, 5–17 (2007).
71. Kaufman, A. J. *et al.* Late Archean Biospheric Oxygenation and Atmospheric Evolution. *Science (80-.).* **317**, 1900–1903 (2007).
72. Kitayama, Y., Thomassot, E., O'Neil, J. & Wing, B. A. Sulfur- and oxygen-isotope constraints on the sedimentary history of apparent conglomerates from the Nuvvuagittuq Greenstone Belt (Nunavik, Québec). *Earth Planet. Sci. Lett.* **355**–**356**, 271–282 (2012).
73. Kurzweil, F. *et al.* Atmospheric sulfur rearrangement 2.7 billion years ago: Evidence for oxygenic photosynthesis. *Earth Planet. Sci. Lett.* **366**, 17–26 (2013).
74. Li, J. *et al.* Primary multiple sulfur isotopic compositions of pyrite in 2.7 Ga shales from the Joy Lake sequence (Superior Province) show felsic volcanic array-like signature. *Geochim. Cosmochim. Acta* **202**, 310–340 (2017).
75. Liu, L., Ireland, T. & Holden, P. In-situ quadruple sulfur isotopic compositions of pyrites in the ca. 3.2–2.72 Ga metasedimentary rocks from the Pilbara Craton, Western Australia. *Chem. Geol.* **557**, 119837 (2020).
76. Mishima, K. *et al.* Multiple sulfur isotope geochemistry of Dharwar Supergroup, Southern India: Late Archean record of changing atmospheric chemistry. *Earth Planet. Sci. Lett.* **464**, 69–83 (2017).
77. Mojzsis, S. J., Coath, C. D., Greenwood, J. P., McKeegan, K. D. & Harrison, T. M. Mass-independent isotope effects in Archean (2.5 to 3.8 Ga) sedimentary sulfides determined by ion microprobe analysis. *Geochim. Cosmochim. Acta* **67**, 1635–1658 (2003).
78. Muller, É. *et al.* Primary sulfur isotope signatures preserved in high-grade Archean barite deposits of the Sargur Group, Dharwar Craton, India. *Precambrian Res.* **295**, 38–47 (2017).
79. Nabhan, S., Marin-Carbonne, J., Mason, P. R. D. & Heubeck, C. In situ S-isotope compositions of sulfate and sulfide from the 3.2 Ga Moodies Group, South Africa: A record of oxidative sulfur cycling. *Geobiology* **18**, 426–444 (2020).
80. Ohmoto, H., Watanabe, Y., Ikemi, H., Poulson, S. R. & Taylor, B. E. Sulphur isotope evidence for an oxic Archaean atmosphere. *Nature* **442**, 908–911 (2006).
81. Ono, S., Kaufman, A. J., Farquhar, J., Sumner, D. Y. & Beukes, N. J. Lithofacies control on multiple-sulfur isotope records and Neoarchean sulfur cycles. *Precambrian Res.* **169**, 58–67 (2009).
82. Ono, S., Beukes, N. J. & Rumble, D. Origin of two distinct multiple-sulfur isotope compositions of pyrite in the 2.5 Ga Klein Naute Formation, Griqualand West Basin, South Africa. *Precambrian*

Res. **169**, 48–57 (2009).
83. Paris, G., Adkins, J. F., Sessions, A. L., Webb, S. M. & Fischer, W. W. Neoarchean carbonate-associated sulfate records positive δ33S anomalies. *Science (80-.).* **346**, 739–741 (2014).
84. Paris, G. *et al.* Deposition of sulfate aerosols with positive Δ33S in the Neoarchean. *Geochim. Cosmochim. Acta* **285**, 1–20 (2020).
85. Papineau, D. & Mojzsis, S. J. Mass-independent fractionation of sulfur isotopes in sulfides from the pre-3770 Ma Isua Supracrustal Belt, west Greenland. *Geobiology* **4**, 227–238 (2006).
86. Papineau, D., Mojzsis, S. J., Coath, C. D., Karhu, J. A. & McKeegan, K. D. Multiple sulfur isotopes of sulfides from sediments in the aftermath of Paleoproterozoic glaciations. *Geochim. Cosmochim. Acta* **69**, 5033–5060 (2005).
87. Papineau, D., Mojzsis, S. J. & Schmitt, A. K. Multiple sulfur isotopes from Paleoproterozoic Huronian interglacial sediments and the rise of atmospheric oxygen. *Earth Planet. Sci. Lett.* **255**, 188–212 (2007).
88. Partridge, M. A., Golding, S. D., Baublys, K. A. & Young, E. Pyrite paragenesis and multiple sulfur isotope distribution in late Archean and early Paleoproterozoic Hamersley Basin sediments. *Earth Planet. Sci. Lett.* **272**, 41–49 (2008).
89. Philippot, P., Van Zuilen, M. & Rollion-Bard, C. Variations in atmospheric sulphur chemistry on early Earth linked to volcanic activity. *Nat. Geosci.* **5**, 668–674 (2012).
90. Philippot, P. *et al.* Response to comment on ‘early archaean microorganisms preferred elemental sulfur, not sulfate’. *Science (80-.).* **319**, 1534–1538 (2008).
91. Roerdink, D. L., Mason, P. R. D., Whitehouse, M. J. & Brouwer, F. M. Reworking of atmospheric sulfur in a Paleoarchean hydrothermal system at Londozi, Barberton Greenstone Belt, Swaziland. *Precambrian Res.* **280**, 195–204 (2016).
92. Roerdink, D. L., Mason, P. R. D., Farquhar, J. & Reimer, T. Multiple sulfur isotopes in Paleoarchean barites identify an important role for microbial sulfate reduction in the early marine environment. *Earth Planet. Sci. Lett.* **331**–**332**, 177–186 (2012).
93. Roerdink, D. L., Mason, P. R. D., Whitehouse, M. J. & Reimer, T. High-resolution quadruple sulfur isotope analyses of 3.2Ga pyrite from the Barberton Greenstone Belt in South Africa reveal distinct environmental controls on sulfide isotopic arrays. *Geochim. Cosmochim. Acta* **117**, 203–215 (2013).
94. Scott, C. *et al.* Pyrite multiple-sulfur isotope evidence for rapid expansion and contraction of the early Paleoproterozoic seawater sulfate reservoir. *Earth Planet. Sci. Lett.* **389**, 95–104 (2014).
95. Shen, Y., Farquhar, J., Masterson, A., Kaufman, A. J. & Buick, R. Evaluating the role of microbial sulfate reduction in the early Archean using quadruple isotope systematics. *Earth Planet. Sci. Lett.* **279**, 383–391 (2009).
96. Slotznick, S. P. *et al.* Reexamination of 2.5-Ga “whiff” of oxygen interval points to anoxic ocean before GOE. *Sci. Adv.* **8**, (2022).
97. Teles, G. S., Chemale, F., Ávila, J. N. & Ireland, T. R. The Paleoarchean Northern Mundo Novo Greenstone Belt, São Francisco Craton: Geochemistry, U–Pb–Hf–O in zircon and pyrite δ34S-Δ33S-Δ36S signatures. *Geosci. Front.* 101252 (2021). doi:10.1016/j.gsf.2021.101252
98. Teles, G. S. *et al.* Textural and geochemical investigation of pyrite in Jacobina Basin, São Francisco Craton, Brazil: Implications for paleoenvironmental conditions and formation of pre-GOE metaconglomerate-hosted Au-(U) deposits. *Geochim. Cosmochim. Acta* **273**, 331–353 (2020).
99. Thomazo, C., Ader, M., Farquhar, J. & Philippot, P. Methanotrophs regulated atmospheric sulfur isotope anomalies during the Mesoarchean (Tumbiana Formation, Western Australia). *Earth Planet. Sci. Lett.* **279**, 65–75 (2009).
100. Thomazo, C., Nisbet, E. G., Grassineau, N. V., Peters, M. & Strauss, H. Multiple sulfur and carbon isotope composition of sediments from the Belingwe Greenstone Belt (Zimbabwe): A biogenic

methane regulation on mass independent fractionation of sulfur during the Neoarchean? *Geochim. Cosmochim. Acta* **121**, 120–138 (2013).

101. Ueno, Y., Ono, S., Rumble, D. & Maruyama, S. Quadruple sulfur isotope analysis of ca. 3.5 Ga Dresser Formation: New evidence for microbial sulfate reduction in the early Archean. *Geochim. Cosmochim. Acta* **72**, 5675–5691 (2008).
102. Wacey, D., McLoughlin, N., Whitehouse, M. J. & Kilburn, M. R. Two coexisting sulfur metabolisms in a ca. 3400 Ma sandstone. *Geology* **38**, 1115–1118 (2010).
103. Wacey, D. *et al.* Geochemistry and nano-structure of a putative ~3240 million-year-old black smoker biota, Sulphur Springs Group, Western Australia. *Precambrian Res.* **249**, 1–12 (2014).
104. Wacey, D., Noffke, N., Cliff, J., Barley, M. E. & Farquhar, J. Micro-scale quadruple sulfur isotope analysis of pyrite from the ~3480Ma Dresser Formation: New insights into sulfur cycling on the early Earth. *Precambrian Res.* **258**, 24–35 (2015).
105. Whitehouse, M. J., Kamber, B. S., Fedo, C. M. & Lepland, A. Integrated Pb- and S-isotope investigation of sulphide minerals from the early Archaean of southwest Greenland. *Chem. Geol.* **222**, 112–131 (2005).
106. Zerkle, A. L., Claire, M. W., Domagal-Goldman, S. D., Farquhar, J. & Poulton, S. W. A bistable organic-rich atmosphere on the Neoarchaean Earth. *Nat. Geosci.* **5**, 359–363 (2012).
107. Zhelezinskaia, I., Kaufman, A. J., Farquhar, J. & Cliff, J. Large sulfur isotope fractionations associated with Neoarchean microbial sulfate reduction. *Science (80-.).* **346**, 742–744 (2014).
