# Supplementary material for: Reconciling discrepant minor sulfur isotope records of the Great Oxidation Event
Source: Nat Commun. 2023 Jan 17;14:279. doi: 10.1038/s41467-023-35820-w (PMC9845385; doi:10.1038/s41467-023-35820-w)
Supplement: Supplementary file 3 — Description of Additional Supplementary Files [file 41467_2023_35820_MOESM3_ESM.pdf]

Description of Additional Supplementary Files:

Supplementary Data File 1: Database compilation of multiple sulphur isotope data used in this study.

Supplementary Data File 2: Compilation of quadruple sulphur isotope data generated for this study.

Supplementary HTML Interface: Plot of the geological Δ33S record through time. Data are colored by phase of sulfur being measured, with the Sulfide and Sulfate phases being further subdivided into Bulk and Spot (e.g. SIMS) analysis types. Clicking on a legend entry will remove data of that phase from the plot, while double clicking an entry will remove all other legend entries. Clicking and dragging within the plot bounds will zoom in on an interval of interest. Clicking the small house symbol in the upper right tool bar will reset the axes. Hovering over a specific data point will bring up a text box with compiled metadata for that analysis, while clicking on a data point will open a new browser tab with a google scholar link to the source of that data. Figure was generated using the plotly package for R (click top rightmost icon for details)
